# Identification of Pathogenicity-Related Effector Proteins and the Role of Piwsc1 in the Virulence of *Penicillium italicum* on Citrus Fruits

**DOI:** 10.3390/jof8060646

**Published:** 2022-06-20

**Authors:** Xiaoying Li, Shuzhen Yang, Meihong Zhang, Yanting Yang, Litao Peng

**Affiliations:** College Key Laboratory of Environment Correlative Dietology, Ministry of Education, College of Food Science and Technology, Huazhong Agricultural University, Wuhan 430070, China; lxying356@163.com (X.L.); zmh1576848384@163.com (M.Z.); 18854802695@163.com (Y.Y.)

**Keywords:** *Penicillium italicum*, host–pathogen interaction, effector, gene knockout, *Piwsc1*

## Abstract

Blue mold caused by *Penicillium italicum* is one of the two major postharvest diseases of citrus fruits. The interactions of pathogens with their hosts are complicated, and virulence factors that mediate pathogenicity have not yet been identified. In present study, a prediction pipeline approach based on bioinformatics and transcriptomic data is designed to determine the effector proteins of *P. italicum*. Three hundred and seventy-five secreted proteins of *P. italicum* were identified, many of which (29.07%) were enzymes for carbohydrate utilization. Twenty-nine candidates were further analyzed and the expression patterns of 12 randomly selected candidate effector genes were monitored during the early stages of growth on PDA and infection of Navel oranges for validation. Functional analysis of a cell wall integrity-related gene *Piwsc1,* a core candidate, was performed by gene knockout. The deletion of *Piwsc1* resulted in reduced virulence on citrus fruits, as presented by an approximate 57% reduction in the diameter of lesions. In addition, the mycelial growth rate, spore germination rate, and sporulation of Δ*Piwsc1* decreased. The findings provide us with new insights to understand the pathogenesis of *P. italicum* and develop an effective and sustainable control method for blue mold.

## 1. Introduction

Citrus fruits are one of the most important commercial fruits with an annual yield over 124.3 million tons worldwide [1]. Postharvest citrus fruits are extremely susceptible to fungal infection, particularly during storage and transportation [2]. In China, citrus blue mold caused by *Penicillium italicum* results in about a 30–50% loss of annual citrus production [3]. The use of chemical fungicides, such as prochloraz and imazalil, has been the primary approach to control blue mold in citrus fruits [4]. However, extensive or intensive applications of these chemicals leads to the emergence of resistant fungal populations and poses risks to human health and the environment [5]. Although biological control has been proposed as an alternative strategy, the efficacy does not currently meet the commercial demands. In addition, the possible action modes of those biological agents have not been fully elucidated [6]. Therefore, a further exploration of the molecular mechanism underlying the interactions between *P. italicum* and citrus fruits is crucial for the development of novel strategies to control citrus blue mold.

During the long-term arms race between pathogens and hosts, plants have developed two sophisticated layers of immune systems for many pathogens: pathogen-associated molecular pattern (PAMP)-triggered immunity (PTI) and effector-triggered immunity (ETI) [7]. In order to escape plant immune system, pathogens are forced to secrete effectors for a successful colonization in the hosts [8]. These effectors can be secreted proteins or small molecules, most of which serve as virulence factors, PAMPs, toxin proteins, elicitors, or degrading enzymes [9]. From a function point of view, effectors can be divided into two categories: one group consists of virulence factors and toxins that allow for the infection and progress of disease, including necrosis and ethylene-induced proteins (NEPs), cerato-platanin proteins, and lysin motifs proteins (LysMs) [10,11,12,13]. The other group containing avirulence factors and elicitors would help fungal defense from plants, which can be represented by effectors AvrPik, Avr1-CO39, AvrPia, and AvrPi-ta in *Magnaporthe oryzae* and elicitor BcCrh1 in *Botrytis cinerea* [14,15,16]. These effector proteins have been found in a variety of pathogens, including *Penicillum* spp. For example, PeNLP1 and PeLysMs produced by *Penicillium expansum* are the virulence effectors for the decay of apple fruits [12,13]. The activation of effector proteins CAZymes (carbohydrate-active enzymes) and SSCPs (small secreted cysteine-rich proteins) was regarded as an important strategy for *P. italicum* colonization on Valencia oranges [17].

The functional characterization of effector proteins of *P. italicum* is an ongoing task. The identification of effector proteins at a molecular level is the premise, but is still challenging due to the lack of uniformity in sequencing data. Similar to other fungal species, the predication of effector proteins in *P. italicum* could also follow broad criteria [18]. In general, effector proteins have the following characteristics [19]: having a signal peptide for secretion; having no transmembrane domain and no glycosylphosphatidylinositol anchor site; being less than 300 amino acid residues in length and rich in cysteine; and being highly expressed in planta. The screening and identification of effector proteins are usually performed by bioinformatic approaches [19,20], in which EffectorP-fungi software is powerful when combined with in planta expression data for predicting effector candidates [21]. The secreted proteins encoded by the genome of sequenced fungi can be analyzed by SignalP, TMHMM, and TargetP servers and then screened based on the characteristics described above, such as the number of amino acid residues and cysteine content. Finally, the selected candidate effectors can be hierarchically clustered [22,23]. With this pipeline approach, Levin et al. [24] predicted 17 candidate proteins that are related to the pathogenesis of *P. expansum*. To date, the characterizations of the effector proteins in plant pathogenic fungi are mainly focused on the fungi that are available for whole genomic sequencing, for instance, *Cladosporium fulvum*, *M. oryzae*, *Fusarium oxysporum*, *Leptosphaeria maculans*, and *P. expansum* [24,25,26,27,28]. These studies have laid a solid foundation for us to study the candidate effectors of *P. italicum* during infection.

In the present study, transcriptomic data from *P. italicum*-infected Navel orange tissues at 72 h post-inoculation (hpi) are used to follow a pipeline search for pathogenic effectors in the whole genome of *P. italicum*. The gene expression patterns of candidate effectors are analyzed under the conditions of in vitro culture and fruit inoculation. One of the five core candidate effectors Piwsc1, a cell wall integrity-related protein, is characterized and functionally analyzed by gene knockout. The findings provide us with new insights to understand the pathogenesis of *P. italicum* and develop an effective and sustainable control method for blue mold.

## 2. Materials and Methods

### 2.1. Fruits and Fungal Cultures

Navel orange fruits (*Citrus sinensis* L. Osbeck) were harvested from an orchard in Ganzhou City, China. The fruits were surface-disinfected with 0.2% sodium hypochlorite for 2 min, then rinsed with tap water, and air-dried before experiments.

*Penicillium italicum* strain was isolated from infected citrus fruits with the typical blue mold symptom. The isolate was confirmed on the basis of the morphological characteristics of the colony. *P. italicum* was cultured on potato dextrose agar (PDA) or in potato dextrose broth (PDB) at 26 °C in darkness. Conidia were collected as previously described by Yang et al. [29].

### 2.2. Secretome Prediction and Annotation

With reference to the workflow of predicting *P. expansum* secreted proteins by Chen et al. [13], an approach was designed to predict the secretome of *P. italicum* with minor modifications. The protein sequences of *P. italicum* (*P. italicum* PHI-1, Accession: GCA_000769765.1) were downloaded from the National Center for Biotechnology Information (NCBI) database (https://www.ncbi.nlm.nih.gov/assembly/GCA_000769765.1, accessed on 15 July 2020) [30]. The following software was used for secretome mining: SignalP (v5.0; http://www.cbs.dtu.dk/services/SignalP/index.php, accessed on 18 July 2020) that was used to determine the presence of signal peptides [31], TargetP (v1.1; http://www.cbs.dtu.dk/services/TargetP/, accessed on 22 July 2020) for subcellular location prediction (Loc = S and Reliability Class ≤ 2) [32], TMHMM (v2.0; http://www.cbs.dtu.dk/services/TMHMM/, accessed on 22 July 2020) for transmembrane (TM) helices prediction (TM ≤ 1) [33], and PredGPI (http://gpcr.biocomp.unibo.it/predgpi/pred.html, accessed on 22 July 2020) was used to remove glycophosphatidylinositol (GPI)-anchored proteins [34]. Finally, the gene function of the secreted proteins was annotated by the Uniprot (https://www.uniprot.org/, accessed on 27 July 2020) database [35] and the protein family classification was predicted by the Pfam (v27.0; http://pfam.xfam.org/, accessed on 27 July 2020) database [36]. CAZyme was annotated by the dbCAN database (http://bcb.unl.edu/dbCAN2/blast.php, accessed on 27 July 2020) combined with HMMER (E-Value < 1 × 10^−15^, coverage > 0.35), DIAMOND (E-Value < 1 × 10^−102^), and Hotpep (Frequency > 2.6, Hits > 6) [37]. 

### 2.3. Sample Preparation, RNA-Seq Library Construction, and Illumina Sequencing

Two wounds (1 × 2 mm) were created uniformly at the equator of the citrus fruit with a 0.5 mm diameter needle. A 10 μL conidia suspension of *P. italicum* (1 × 10^6^ spores mL^−1^) was injected into each wound. The inoculated fruits were placed in transparent sealed boxes and stored at 26 °C. The tissue samples (about 3 g) were collected from the wound after 72 hpi. They were immediately frozen with liquid nitrogen and stored at −80 °C for subsequent analysis. Three replicates were prepared for the sample. RNA was extracted using a plant total RNA extraction kit (Aidlab Biotechnologies Co., Ltd., Beijing, China), according to the manufacturer’s instructions (with three biological replicates). The Illumina HiSeq 2000 sequencing platform was used for sequencing total RNA, which was conducted by the BGI Company (Shenzhen, China). The raw RNA-Seq data files were submitted to the NCBI database under SRA accession number SRP362092.

### 2.4. Effector Protein Prediction Pipeline

Based on the results of secretome prediction and RNA-seq data, secreted proteins highly induced by infected Navel oranges were selected with an expression level greater than an average expression level (the average FPKM value of all genes detected in the genome of *P. italicum*) [24]. According to the method of Levin et al. [24], with slight modifications, the highly induced secreted proteins were classified according to the following criteria: (a) small (<400 aa) and cysteine-rich (>4) proteins; (b) proteins with gene function annotations or belonging to known protein families; (c) proteins predicted to be effectors using EffectorP-fungi (v3.0; http://effectorp.csiro.au/, accessed on 30 September 2020) software [21]; and (d) proteins belonging to known effector families or with effector domains that have been previously reported in the literature. Proteins fulfilling two or more of the above characteristics were selected as the optimal candidate effectors.

### 2.5. RNA Extraction and cDNA Synthesis

The tissue samples from the wounded sites of Navel oranges were collected at 24, 48, and 72 hpi using 0 hpi as a control. The mycelium samples from PDA medium at 26 °C were collected at 24, 48, 72, and 96 hpi, respectively. *P. italicum* spores that were frozen immediately after culturing on PDA medium at 26 °C for one week were prepared as a control. 

The total RNA of *P. italicum* mycelia and Navel orange tissues were extracted using an RNA kit (Omega Bio-tek, Doraville, GA, USA) and plant total RNA extraction kit, respectively, according to the manufacturer’s instructions. cDNA was synthesized with a HiScript ^®^II Q RT SuperMix for qPCR (+gDNA wiper) reverse transcription kit (Vazyme Biotechnology Co., Ltd., Nanjing, China), following the manufacturer’s protocols. Each experiment was repeated three times. 

### 2.6. PCR and Reverse Transcription-Quantitative PCR (RT-qPCR)

PCR reactions were performed using a standard thermocycler (Bio-rad T100, Hercules, CA, USA), according to the manufacturer’s protocols. Each 25 μL of PCR reaction contained 2.5–5 U EasyTaq DNA polymerase (Transgen Biotech, Beijing, China), 10× Ex Taq buffer, 0.2 mM dNTPs (2.5 mM), 0.2 μM of each primer, and 1 ng-1μg template DNA. PCR conditions were 5 min at 94 °C, followed by 30 cycles of 30 s at 94 °C, 30 s at 56–60 °C, 1–2 kb/min at 72 °C, and 10 min at 72 °C. Primer sequences are listed in Table S1.

RT-qPCR was performed with a CFX96 Real-Time PCR Detection System (Analytik Jena, Germany), according to the manufacturer’s protocols. Each 25 μL of PCR reaction contained 5 μL of ChamQ SYBR qPCR Master Mix (Vazyme Biotechnology Co., Ltd., Nanjing, China), 1 ng-100 ng cDNA, and 0.2 μM of each primer. The reaction procedure was 95 °C 30 s, followed by 40 cycles of 5 s at 95 °C, 30 s at 60 °C; melt curve. Samples collected at 0 hpi were used to calibrate expression levels. The *β-actin* gene of *P. italicum* was used as an internal reference [17]. A combination of three biological replicates with three technical replicates of each reaction were used. The relative expression levels of genes were calculated by the 2^−ΔΔCt^ method [38].

### 2.7. Piwsc1 Sequence and Phylogenetic Analysis

The conserved domains of *P. italicum* PiWSCs were analyzed by InterPro (v5.1; http://www.ebi.ac.uk/interpro/, accessed on 29 March 2022) [39]. To establish the evolutionary relationship of *P. italicum* Piwsc1, the amino acid sequence of *P. italicum* Piwsc1 was analyzed by BLASTp, and 20 species from 10 genera were selected; then the phylogenetic tree of *P. italicum* Piwsc1 was constructed with MEGA11 software using the Neighbor Joining method [40]. The bootstrap consensus tree was inferred from 1000 replicates. 

### 2.8. Construction and Verification of Piwsc1 Knockout and Complementation Mutant 

Gene disruption by homologous recombination was performed, as described earlier by Catlett et al. [41]. For *Piwsc1* knockout, flanking regions of the 5′-region (1467 bp) and 3′-region (1281 bp) of the *Piwsc1* gene (PITC_087410) were PCR amplified from the genomic DNA of wild-type *P. italicum* (WT) using wsc1up-F/R and wsc1do-F/R primer pairs, respectively (Table S2). Figure S1a presents the knockout cassette construction strategy. The knockout box was constructed by the fusion of the 5′-region, resistance gene *HygB*, and 3′-region, and connected to the pMD19-T vector to obtain the pMD-*Piwsc1* plasmid. Firstly, the pMD-*Piwsc1* vector and pCAMBIA3300 plasmid were digested with *SmaI* and *HindIII*, then ligated with T4 ligase and transformed into *Escherichia coli* DH5α to obtain the *Piwsc1* gene knockout vector. 

*Piwsc1* complementation mutants were constructed using the protocol previously described by Yang et al. [11] with slight modifications. The full-length *Piwsc1* gene was amplified from WT genomic DNA. The fragments of the 5′-region (wsc1up5-F/R, 1137 bp), resistance gene *G418* (G418-F/R, 1221 bp), *TrpC* (TrpC-F/R, 368 bp), and *Piwsc1*+3′-region (wsc1-F/wsc1do3-R, 1984 bp) were fused to construct the complementation box (Figure S1b), and then the complementation vector of the *Piwsc1* gene was obtained according to the process of constructing a knockout vector.

The selected vectors were introduced into *Agrobacterium tumefaciens* AGL-1 cells, which were subsequently used to transform *P. italicum*, as previously described [42]. The obtained transformants were screened for resistance using the corresponding antibiotics (knockout transformants: 50 µg/mL hygromycin B/complementation transformants: 30 µg/mL geneticin). Select positive transformants to extract genomic DNA by the CTAB method [43], and use specific detection primers for PCR and RT-PCR identification (Table S2). 

### 2.9. Phenotype of the Knockout Transformant (ΔPiwsc1) and Complementation Transformant (ΔPiwsc1-co)

Radial growth was examined following the protocol described by Yang et al. [29]. The fungus disc was made with a 5 mm punch and inoculated onto a PDA plate. The radial growth of the cultures was measured daily. Spore germination was evaluated by the calculation of percent germination using a ZEISS microscope (Zeiss Co., Oberkochen, Germany). Microscope images of the germinating spores were obtained following 10 h of incubation at 26 °C of 10 µL spore suspension (3 × 10^6^ spores mL^−1^) inoculated on 0.5% agar-PDA plates. Sporulation was determined according to Levin et al. [24]. A total of 3 mL of sterile distilled water was added to three-days-old PDA plates; each plate was washed with an additional 2 mL of distilled water to ensure maximum spore collection. The resulting spore suspensions were diluted and counted with a hemocytometer. The number of spores per plate was calculated as spore concentration (spores mL^−1^) × dilution factor × 5 mL.

### 2.10. Virulence Assay

The virulence assay was performed according to Yang et al. [44]. The fruits were inoculated with *P. italicum* as described above. Five Navel oranges constituted a biological replicate and three replicates were used for each treatment. The average decay diameters of each treatment were monitored at 3, 4, 5, and 6 d post-inoculation (dpi).

### 2.11. Statistical Analysis

All statistical analyses were performed by one-way analysis of variance (ANOVA) using SPSS 22.0 statistical software (SPSS Inc., Chicago, IL, USA). Mean separations were performed by Fisher’s least significant difference (LSD) test (*p* < 0.05). Different letters indicated significant difference. Bars indicated standard error.

## 3. Results

### 3.1. Prediction of P. italicum Secretome

**Figure 1:**
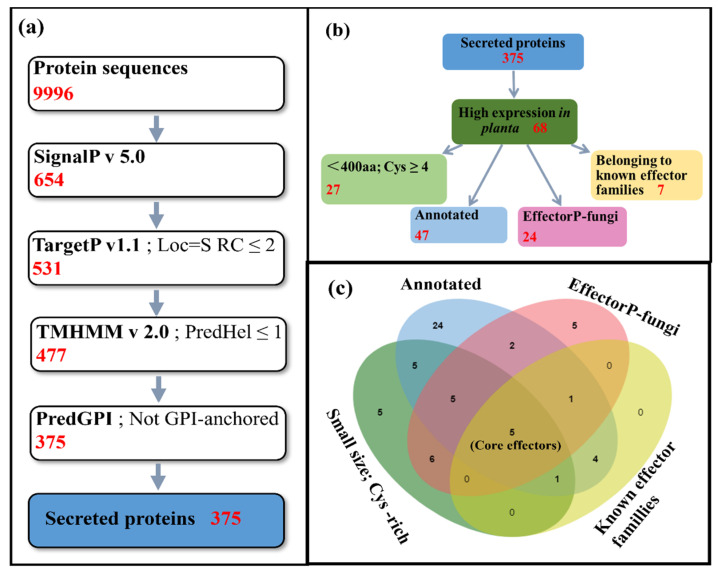
*Penicillium italicum* secretory protein prediction pipeline (**a**), effector protein prediction pipeline (**b**), and Venn analysis (**c**). In (**c**), 29 optimal candidate effector proteins, with five out of them being core candidate effector proteins, are identified. Green: proteins smaller than 400 amino acids with 4 or more cysteines; blue: proteins with gene function annotation or belonging to known protein families; pink: proteins predicted to be effectors by EffectorP-fungi; yellow: proteins belonging to known effector families or with effector domains that have been previously reported in the literature.

The genome sequence and annotation of the *P. italicum* strain PHI-1 was previously described by Ballester et al. [30]. The genome contains a total of 9996 protein sequences, which were used to predict and identify secreted proteins. SignalP v5.0, TargetP v1.1, TMHMM v2.0, and PredGPI were collectively employed to predict the total secretome (Figure 1a, Table S3). Six hundred and fifty-four proteins were identified with signal peptides by analyzing the total protein sequences through SignalP v5.0. After excluding the proteins containing either a chloroplast transit peptide or a mitochondrial targeting peptide by TargetP v1.1, 531 proteins were obtained with extracellular localization signals, of which 477 proteins were selected by TMHMM v2.0 analysis that had no or only one harbor transmembrane helix. Using PredGPI to predict the proteins that harbor glycophosphatidylinositol anchor motifs, 102 proteins were excluded as surface proteins rather than secreted effectors. Eventually, a total of 375 secreted proteins were selected for further analysis, representing 3.8% of the *P. italicum* proteome. 

### 3.2. CAZymes of P. italicum Secretome

**Figure 2:**
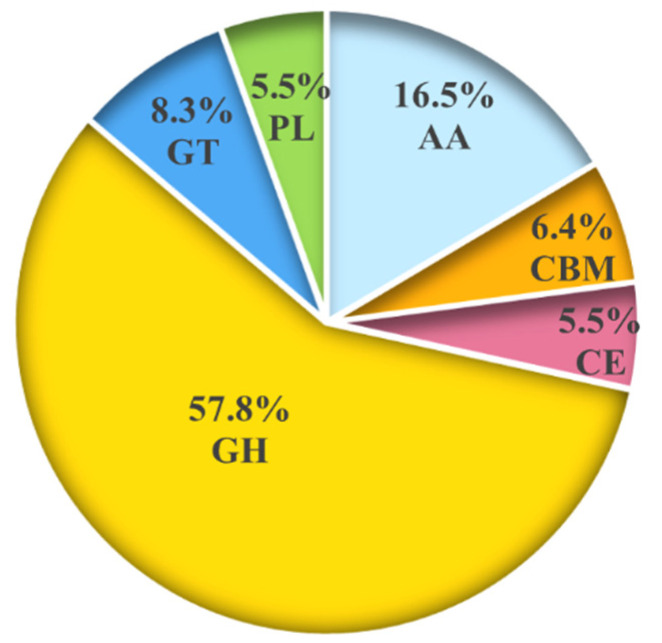
Analysis of six types of carbohydrate-active enzymes (CAZymes) in the secreted proteins of *P. italicum.* GHs: glycoside hydrolases, CEs: carbohydrate esterases, PLs: polysaccharide lyases, GTs: glycosyltransferases, AAs: auxiliary module enzymes, CBMs: carbohydrate-binding modules.

CAZymes play important roles in the pathogenicity of pathogenic fungi [45]. Among the 375 secreted proteins, 109 secreted proteins (29.07%) belonging to the CAZymes family were classified in 58 CAZymes subgroups by using the dbCAN database. Most of them are glycoside hydrolases (GHs), accounting for 57.8% (Figure 2, Table S4). In the current study, seven carbohydrate-binding proteins that have been annotated as concanavalin A-like lectins (GH7, GH11, GH16, GH54) were identified as secreted proteins during the invasion of *Sclerotinia sclerotiorum* on rape [46]. In addition, three secreted proteins that are associated with cell-wall-degrading enzymes, including pectinesterase (CE8) and fungal chitosanase (GH75), are also included in the category of CAZymes.

### 3.3. Analysis of P. italicum Candidate Effector Proteins

We used the commercial platform to establish the RNA-seq library. RNA-seq experiments were conducted using interface tissues from wild-type *P. italicum*-infected Navel oranges. The number of expressed genes detected on the Illumina HiSeq sequencing platform was 8766. The FPKM values of 375 secreted proteins in RNA-Seq are listed in Table S5.

Based on the predicted secreted proteins of *P. italicum*, we screened the genes that were highly expressed on infected Navel oranges, resulting in 68 genes in our prediction pipeline (Figure 1b). The highly expressed secreted proteins were classified as follows. 

#### 3.3.1. Small and Cysteine-Rich Highly Expressed Secreted Proteins

When screening highly expressed secreted proteins by length and the number of cysteine residues, 27 secreted proteins matched the conditions of small-sized (<400 aa) and cysteine-rich (>4) (Table S6). Half of these proteins were 100–200 amino acid residues in length and most of the effector proteins had 4 and 6 cysteine residues.

#### 3.3.2. Highly Expressed Secreted Proteins with Functional Annotations or Belonging to Known Protein Families

Forty-seven secreted proteins have functional annotations or belong to known protein families. Among them, 11 secreted proteins belong to known effector families or contain domains that have been found in fungal effectors, which include cell wall integrity and the stress response component (WSC) domain (PF01822) [47], concanavalin A-like lectin (PF00457) [46], cerato-platanin (PF07249) [11,48,49], necrosis-inducing protein (NIP) (PF05630) [16], pectin lyase fold/virulence factor (PF00544) [50], ribonuclease (RNase) (PF00545, PF00445) [51,52], and peptidase family (PF00082, PF09286, PF05922, PF05577, PF00450) [24,28,53,54] (Table S7). 

#### 3.3.3. Highly Expressed Secreted Proteins by EffectorP-fungi Software Prediction

Through EffectorP-fungi software, 24 of 68 secreted proteins were found to meet the EffectorP effector protein criteria (Table S8); 15 were predicted as apoplastic effectors, 4 were predicted as cytoplasmic effectors, and 5 were predicted either as apoplastic or cytoplasmic effectors.

As mentioned above, proteins meeting two or more of the above criteria were selected to be analyzed further as optimal candidates (Figure 1c), which resulted in 29 candidate effectors (Table 1). Among them, five annotated proteins belonged to the glycoside hydrolase family (PITC_048860, PITC_077450, PITC_005000, PITC_081180, and PITC_020870). Another five proteins were annotated as peptidase (PITC_061260, PITC_079910, PITC_007270, PITC_014210, and PITC_008610), in which one (PITC_061260) had aspartic-type endopeptidase activity and the other four had a serine-type carboxypeptidase activity. PITC_043760 and PITC_047900 presented RNase activity. Two hydrophobic proteins (PITC_001010 and PITC_015600) were considered to be the structural constituents of the cell wall, and one (PITC_034160) was a pectate lyase. PITC_013620 belonged to the p24 (emp24/gp25L) family of transmembrane proteins. PITC_019680 was annotated as thioredoxin. PITC_081470 belonged to phosphatase. PITC_099400 contained a myeloid differentiation factor 2-related lipid-recognition (ML) domain that was involved in innate immunity process and sterol transport. PITC_097880 encoded a NIP and possessed a nucleotide pyrophosphatase/phosphodiesterase1 (NPP1) domain. PITC_087410 contained a WSC domain that participated in the cell wall integrity (CWI) signaling pathway. PITC_016950 belonged to cerato-platanin proteins. In addition, seven were hypothetical proteins with no functional annotation, but highly expressed during infection in citrus fruits (PITC_045800, PITC_051450, PITC_068580, PITC_014450, PITC_085860, PITC_014290, and PITC_062780). Five of them, namely, carbohydrate-binding WSC (PITC_087410), cerato-platanin (PITC_016950), pectin lyase (PITC_034160), ribonuclease_T2 (PITC_043760), and guanine-specific ribonuclease N1/T1 (PITC_047900), met all of the above criteria, and thus were regarded as core effector proteins (Table 1).

### 3.4. RT-PCR and RT-qPCR Analyses of Candidate Effector Genes

**Figure 3:**
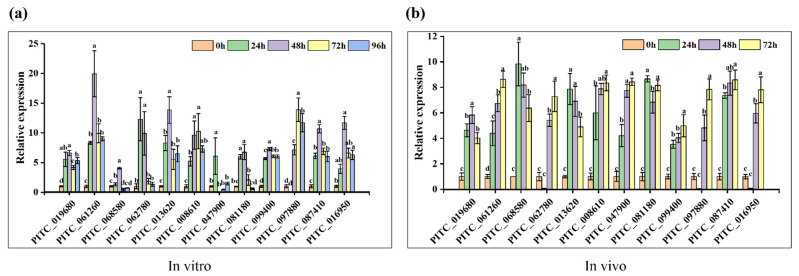
Expression patterns of 12 candidate effector genes are determined by RT-qPCR when cultivated on PDA (in vitro) (**a**) and during infection and development of *P. italicum* on Navel oranges (in vivo) (**b**). *β-actin* gene of *P. italicum* serves as an endogenous control. Samples collected at 0 h post-inoculation (hpi) are used to calibrate expression levels. Different letters indicated significant difference. Bars indicated standard error.

To validate whether these effectors of *P. italicum* were related to the early stage of infection, the time lines of blue mold symptoms and disease development in Navel oranges were monitored at 24, 48, and 72 hpi (Figure 3). At 24 hpi, the wound sites of the fruits began to collapse and the wound edges were in a water-soaked soft-rot state. A small amount of mycelium on the soft-rot sites appeared at 48 hpi. The soft rot rapidly expanded by 72 hpi and conidia generated on the mycelia (Figure S2). Symptomatically, time points between 48 hpi and 72 hpi seemed to be critical for *P. italicum* infection.

The time course of expression patterns of twenty-nine candidate genes were studied using RT-PCR with total RNAs extracted from *P. italicum*-infected Navel oranges at 0, 24, 48, and 72 hpi (Figure S3). A total of 24 effector genes were detected at 24 hpi, indicating that they might be involved in the early process of *P. italicum* for invasion and colonization on citrus fruits. The remaining 5 candidate effector genes (PITC_051450, PITC_085860, PITC_001010, PITC_015600, and PITC_097880) began to express at 48 hpi, which might be required for a late pathogenicity. 

Twelve randomly selected candidate effector genes were further investigated by RT-qPCR for their expression on PDA culture (in vitro) (Figure 3a) and during Navel orange infection (in vivo) (Figure 3b). We found that all 12 candidate effector genes were actively expressed both in vitro and in vivo. On the PDA culture, 9 effector genes exhibited the highest expression levels at 48 hpi, with 2 effector genes (PITC_062780 and PITC_047900) reaching peaks at 24 hpi and 1 effector gene (PITC_097880) at 72 hpi. During Navel orange infection, the expression patterns of 8 effector genes demonstrated an upward tendency, with 3 effector genes (PITC_068580, PITC_013620, and PITC_081180) reaching their peaks at 24 hpi, and 1 effector gene (PITC_019680) at 48 hpi. However, the expression patterns of effector genes in vitro were not always consistent with those in vivo. For example, the expression of PITC_062780 reached its peak at 24 hpi in vitro, whereas its expression was not detected in vivo at this time. The expression levels of the three selected core effector genes PITC_047900, PITC_087410, and PITC_016950 reached peaks at 24 hpi, 48 hpi, and 48 hpi in vitro, respectively, while their expression patterns exhibited an increasing tendency in vivo. Taken together, these results suggest that these core effectors might play important roles in the pathogenicity of this pathogen.

Based on the above analyses, PITC_087410 (denoted as Piwsc1) matched all the criteria for candidate effector proteins and were higher expressed in vitro (24–96 hpi) and in vivo (24–72 hpi) conditions, which was then selected for further functional analysis.

### 3.5. Characterization of Piwsc1

**Figure 4:**
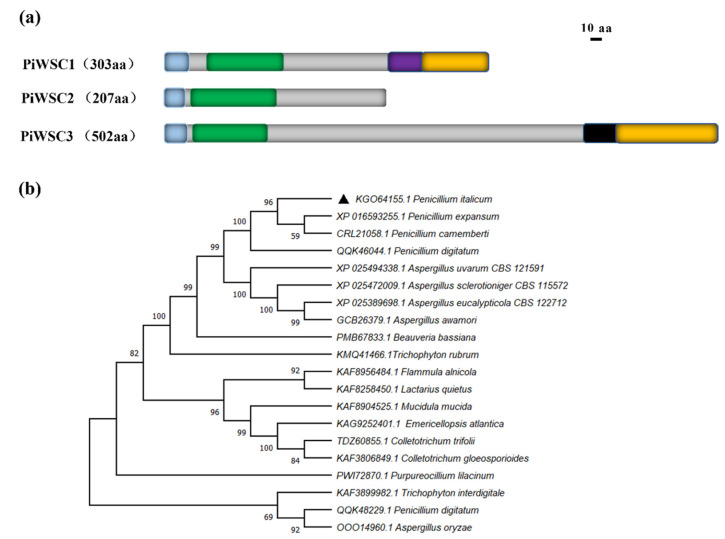
Conserved domains and phylogenetic tree analysis of Piwsc1. (**a**). Prediction of conserved domains of PiWSC protein sequences using InterPro. Diagrammatic representation of the structures of the three PiWSC proteins. Blue rectangles—signal peptides; green rectangles—WSC domains; purple rectangle—SKG6 domain; orange rectangles—cytoplasmic domains; black rectangle—TM_EGFR-like domain. The amino acid sequence length in aa is given for each protein as an indication of scale. (**b**). The evolutionary relationship of Piwsc1 and its homologs from other fungi.

The *Piwsc1* gene encodes a protein of 303 aa residues with the full-length of 976 bp, which contains one intron. Except for Piwsc1, PITC_001070 (denoted as PiWSC2) and PITC_052160 (denoted as PiWSC3) were also annotated as carbohydrate-binding WSC proteins in the genome of *P. italicum* (Figure 4a). PiWSC2 is a GPI-anchored protein, and PiWSC3 shows a reliability score of more than 2 by TargetP analysis; both of them were excluded as secreted proteins. The structural domains analysis by InterPro showed that all three WSC proteins had a signal peptide at the N-terminal, and Piwsc1 and PiWSC3 had a cytoplasmic domain at the C-terminal. In addition, Piwsc1 had an SKG6 domain, and PiWSC3 had a transmembrane domain of the epidermal growth factor receptor family of protein tyrosine kinases (TM_EGFR). By contrast, PiWSC2 did not appear to possess any other conserved domains. The protein sequence alignment results show that the three proteins have a low sequence identity between them (Figure S4).

Piwsc1 is conserved in genus *Penicillium* and other fungi genera. The phylogenetic tree showed that *P. italicum* Piwsc1 had the highest homology with *P. expansum* and a higher sequence identity with the homologues of the *Aspergillus* species than other filamentous fungi (Figure 4b).

### 3.6. Functional Analysis of Piwsc1

To further explore the biological function of *Piwsc1* in *P. italicum*, *Piwsc1* knockout and complementation mutants of *P. italicum* were constructed. The mutants were verified by PCR and RT-PCR using specific primers (Figure S1c–e). A knockout mutant, Δ*Piwsc1*, and a complementation mutant, Δ*Piwsc1-co*, were then selected for functional analysis.

**Figure 5:**
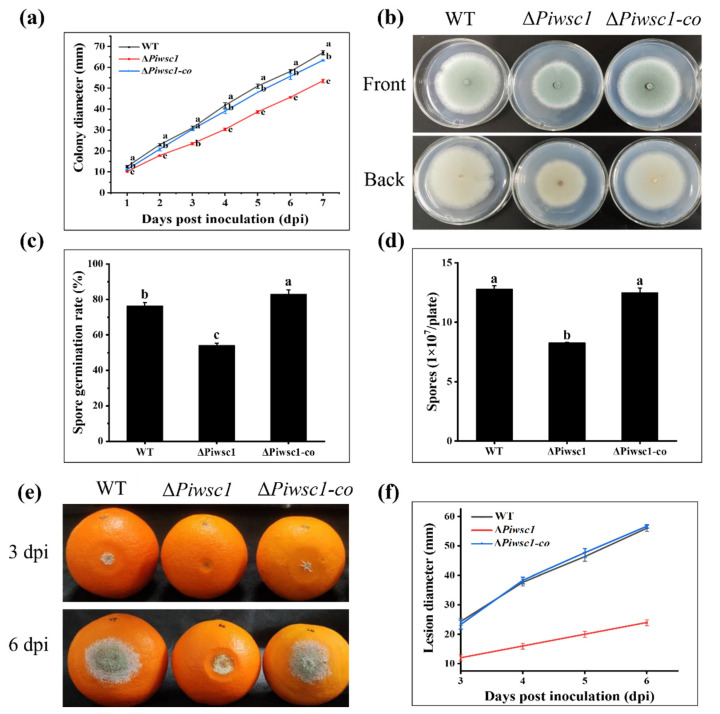
Effect of *Piwsc1* deletion on *P. italicum* radial growth, sporulation, spore germination, and virulence. Radial growth diameter changes (**a**) and colony morphology at 7 dpi (**b**), spore germination rate at 10 hpi (**c**), and sporulation at 3 dpi (**d**) of WT, Δ*Piwsc1*, and Δ*Piwsc1-co* strains on PDA plates are recorded. Rot status at 3 and 6 dpi (**e**) and lesion diameters from 3 to 6 dpi (**f**) of WT, Δ*Piwsc1*, and Δ*Piwsc1-co* strains on Navel oranges are recorded.

Compared to the WT, the radial growth rate of Δ*Piwsc1* was decreased by 20.15% at 7 dpi (Figure 5a). The colony morphology of Δ*Piwsc1* was also altered, as manifested by the decreased hyphal edge density (Figure 5b). The spore germination rate was reduced by 22.27% at 10 hpi (Figure 5c), and the sporulation was reduced by 35.31% (Figure 5d). These findings indicate that *Piwsc1* plays an important role in the growth and development of *P. italicum*.

To determine the role of the *Piwsc1* gene in pathogenicity, Navel oranges were inoculated with WT, Δ*Piwsc1*, and Δ*Piwsc1-co* strains, respectively, and the symptoms of fruit decay were recorded. At the initial stage of infection with Δ*Piwsc1*, the spore formation and the degree of soft rot near the infection site were much lower than WT and Δ*Piwsc1-co* (Figure 5e), and the diameter of citrus lesions inoculated with Δ*Piwsc1* decreased by 57.14%, compared to WT and Δ*Piwsc1-co* at 6 dpi (Figure 5f). The results demonstrate that *Piwsc1* plays an important role in the virulence of *P. italicum.*

## 4. Discussion

Effector proteins are important weapons of pathogenic fungi to compete with plants and cause infection [19,55]. The interaction mechanism between *P. italicum* and citrus fruits at the molecular level was studied to predict and confirm pathogenic effector proteins through bioinformatic analysis, transcriptomic patterns during the infection, and the in vitro growth of deletion mutants from one core gene. A total of 375 secreted proteins of *P. italicum* were predicted by SignalP, TargetP, TMHMM, PredGPI, and other software. Of the screened 29 candidate effector proteins, 5 proteins were finally considered as core effectors that met 4 requirements of the criteria designed in the pipeline. We predicted that secreted proteins accounted for 3.8% of the total proteins in *P. italicum*, which was consistent with reported 3.0%, 4.1%, 4.7%, and 5.1% for *P. infestans*, *Penicillium digitatum_*PHI26, *P. italicum* GL_Gan1, and *S. sclerotiorum*, respectively [17,46,56]. A large portion of secreted proteins in *P. italicum* are enzymes that function as glycosyl hydrolases, proteinases, peptidases, RNases, and pectin lyases (GH55, PL1, and PL3). Pectin lyases were regarded as virulence factors in a number of species due to their cell-wall-degrading activities [57]. These results are consistent with the previous studies predicting that *Fusarium graminearum* secretes various putative enzymes that degrade different components of host cells [58]. Some studies have confirmed that secreted proteins expressed during the infection stage are more likely to be pathogenic effectors [59]. For example, the expression levels of NEP1-like Tal6 and LysM effectors were highly expressed during the early stages of infection caused by various fungi [10,27,60,61]. Notably, some effector genes of plant pathogens seem to be solely expressed during host colonization [62,63], and others, such as PePRT and PeLysMs effectors in *P. expansum,* can be expressed during apple colonization and in vitro growth [24,64]. In this study, while the randomly selected 12 effector genes were actively expressed in vitro and in vivo, we also noted that the induced expression levels in vivo were higher than those in vitro (Figure 3).

Among the 29 effector proteins of *P. italicum*, 18 conserved domains [46] were identified, such as NPP1, WSC, RNase, and cerato-platanin. Protein PITC_097880 was annotated as NIP that contained an NPP1 domain. The most thoroughly studied NIPs were within a family of non-catalytic NIPs. They were collectively named as necrosis- and ethylene-inducing peptide 1-like proteins (NLPs) [16]. NLPs were first identified as the elicitors of cell death in dicotyledonous plants, which might promote plant infection by necrotrophic pathogens and stimulate plant innate immunity [65]. PITC_047900 and PITC_043760 were annotated as RNases, which might be related to pathogenicity since a homology protein—Fg12 from *F. graminearum* contributed to pathogen virulence and induced plant cell death [52]. PITC_016950 belongs to cerato-platanin proteins, a phytotoxic protein secreted by filamentous fungi. In *S. sclerotiorum,* SsCP1 is an important virulence factor and is recognized by plants to trigger plant defense responses [11]. Functional annotations were difficult for 7 effector proteins in this study since no conserved domain was found, although they were highly expressed during the *P. italicum*–citrus interaction. These group of proteins were predicted as apoplastic or cytoplasmic effector proteins on the EffectorP-fungi platform. Chen et al. [66] used the *A. tumefaciens*-mediated *Nicotiana benthamiana* transient expression system to verify the functions of 3 such effectors in *Fusarium sacchari*. The results showed that *Fs*00367 and *Fs*00597 suppressed BAX-induced cell death, while *Fs*05897 induced cell death. This indicated that the hypothetical proteins might also function as effector proteins.

Generally speaking, the low molecular weight and richness of cysteines are two important characteristics of effector proteins, but 7 effector proteins did not accord with these features in our case. For example, the PITC_014210, PITC_008610, PITC_079910, and PITC_007270 encoded serine proteases with a length over 500 amino acids. Many reports have shown that serine proteases play a key role in the pathogenicity of plant pathogens. The first form of evidence that subtilisin (a very diverse family of serine peptidases) is involved in the plant–pathogen interaction was found in tomatoes by Granell et al. [67], who found that subtilisin P69 accumulated in tomato leaves after the tomatoes were treated by citrus exocortis viroid. Furthermore, the *Plasmodiophora brassicae* serine protease Pro1 was identified as a member of the S28 protease family and was previously characterized to play an important role in stimulating dormant spore germination [54]. PITC_020870 was annotated as concanavalin A-like lectin/glucanase with only two cysteines, but it was predicted to be an apoplastic effector (Table S8). Similarly, the AvrLm1 effector of *L. maculans* was localized in the apoplast with only one cysteine [26]. This indicates that the high molecular weight and containing a small number of cysteines may also be an effector protein.

A core candidate effector, Piwsc1, was selected to verify its role in *P. italicum* infection in this study. WSC proteins were regarded as candidate effector proteins in *P. expansum* colonization on apples and identified as secreted proteins of entomopathogenic fungus *Metarhizium anisopliae* when grown under submerged fermentation in the presence of chrysalis as an inducer [24,47]. Similar to homologs in other fungi, the RT-qPCR results exhibited that *Piwsc1* was highly induced during the *P. italicum* infection of citrus fruits. The deletion of Piwsc1 in *P. italicum* affected in vitro growth and infection. The growth rate, spore germination rate, and sporulation of Δ*Piwsc1* were lower than those of WT and Δ*Piwsc1-co*, which are similar to other fungi, such as *Aspergillus nidulans*, in which the growth and sporulation were inhibited after the deletion of homologous protein genes *wscA* and *wscB* [68]. Moreover, the phenotypic changes in Δ*Piwsc1* were mainly characterized by lower mycelial edge density compared to the WT and Δ*Piwsc1-co* strains. Similar results were also shown in *Aspergillus fumigatus* [69]. WSC proteins were described as sensors that were involved in the CWI pathway and stress responses [70,71]. A recent study revealed that proteins containing WSC domains have carbohydrate-binding capacities. In rice blast pathogen *Pyricularia oryzae*, the WSC domain in an alcohol oxidase PoAlcOX directed the attachment to xylan and fungal chitin/β-1,3-glucan [72]. Another example is root endophyte *Serendipita indica* SiWSC3, a lectin-like member transcriptionally induced in planta, binds to long-chain β-1–3-glucan, efficiently agglutinates fungal cells, and is additionally induced during fungus–fungus confrontation [73]. More evidence suggests that WSCs are involved in the processes of host–fungal interactions and fungus–fungus confrontation. Tong et al. [74] found that the deletion of *wsc1I* reduced the conidial infectivity and virulence of entomopathogenic fungi *Beauveria bassiana* to *Galleria mellonella* larvae. Our results for Δ*Piwsc1*-infected Navel oranges also exhibit a significant reduction in virulence, suggesting its active roles in *P. italicum* pathogenesis; however, the underlying mechanism needs further investigation.

## 5. Conclusions

In the current study, the potential effector proteins in *P. italicum*, a common fungal species causing postharvest blue mold of citrus fruits, were characterized. We designed a prediction pipeline to predict the effector proteins of *P. italicum* and used this pipeline to screen out 29 optimal candidate effectors. Our results show that 12 randomly selected candidate effector genes are highly expressed during the early stages of growth on PDA and infection on citrus fruits. Functional analysis was performed on a core candidate effector protein, Piwsc1, and the results demonstrate that the deletion of *Piwsc1* decreases the virulence of this fungus on citrus fruits. Our study on the effector proteins of *P. italicum* provides a theoretical basis for further exploring the molecular mechanism of the citrus–*P. italicum* interaction.

## Figures and Tables

**Table 1:** Twenty-nine candidate effector proteins of *P. italicum*.

Classification	Gene Name	Size (aa)	Cys	Effector Prediction	Function Annotation
Requisite characteristics of (a)*, (b)*, (c)* and (d)*	PITC_087410	303	9	Apoplastic effector	Carbohydrate-binding WSC, subgroup
	PITC_016950	152	4	Apoplastic effector	Cerato-platanin
	PITC_034160	377	6	Apoplastic effector	Pectin lyase fold/virulence factor
	PITC_043760	257	10	Apoplastic effector	Ribonuclease_T2
	PITC_047900	129	4	Apoplastic/cytoplasmic effector	Guanine-specific ribonuclease N1/T1
Requisite characteristics of (a)*, (b)* and (c)*	PITC_001010	117	8	Apoplastic/cytoplasmic effector	Hydrophobin
	PITC_015600	164	8	Apoplastic effector	Hydrophobin
	PITC_005000	368	8	Apoplastic effector	Glycoside hydrolase, family 28
	PITC_081180	374	9	Apoplastic effector	Glycoside hydrolase, family 28
	PITC_099400	174	4	Cytoplasmic effector	MD-2-related lipid recognition
Requisite characteristics of (a)*, (b)* and (d)*	PITC_097880	285	7		Necrosis-inducing protein
	PITC_020870	216	2	Apoplastic effector	Concanavalin A-like lectin/glucanase superfamily, GH11
Requisite characteristics of (a)* and (b)*	PITC_048860	316	4		Glycoside hydrolase, superfamily, GH17
	PITC_019680	367	4		Endoplasmic reticulum, protein ERp29, C-terminal
	PITC_061260	399	4		Peptidase aspartic, catalytic
	PITC_045800	255	4		Hypothetical protein
	PITC_077450	393	10		Glycosyl transferase, family 15
Requisite characteristics of (a)* and (c)*	PITC_051450	95	6	Apoplastic/ cytoplasmic effector	Hypothetical protein
	PITC_068580	103	6	Apoplastic/cytoplasmic effector	Hypothetical protein
	PITC_014450	136	6	Apoplastic/cytoplasmic effector	Hypothetical protein
	PITC_085860	195	6	Apoplastic effector	Hypothetical protein
	PITC_014290	122	7	Apoplastic effector	Hypothetical protein
	PITC_062780	173	8	Apoplastic effector	Hypothetical protein
Requisite characteristics of (b)* and (c)*	PITC_013620	208	2	Cytoplasmic effector	Emp24/gp25L/p24 family/GOLD
	PITC_081470	462	4	Apoplastic effector	Phosphoesterase
Requisite characteristics of (b)* and (d)*	PITC_014210	601	7		Peptidase S8/S53, subtilisin/kexin/sedolisin
	PITC_008610	496	3		Proteinase inhibitor I9
	PITC_079910	517	7		Peptidase S28
	PITC_007270	559	7		Peptidase S10, serine carboxypeptidase

(a)*: proteins smaller than 400 amino acids with 4 or more cysteines; (b)*: proteins with gene function annotations or belonging to known protein families; (c)*: proteins predicted to be effectors using EffectorP-fungi 3.0 software; (d)*: proteins belonging to known effector families or with effector domains that have been previously reported in the literature.

## Data Availability

All the raw RNA-seq data analyzed in this study can be freely downloaded from SRA database in NCBI (https://www.ncbi.nlm.nih.gov/sra/?term=SRP362092, accessed on 22 July 2020), with the sample accession numbers SRX14335696, SRX14335697, and SRX14335698.

## Supplementary Materials

The following supporting information can be downloaded at: https://www.mdpi.com/article/10.3390/jof8060646/s1, Figure S1: Construction and analysis of Δ*Piwsc1* mutants and complementary mutants. Strategy to construct *Piwsc1* knockout (a) and complementation (b) replacement cassettes by double-joint PCR. (c) PCR validation of the knockout strain (Δ*Piwsc1*). Lane Marker, 1 kb marker. Lanes 1 and 2 represent the amplification results of the knockout strain (Δ*Piwsc1*) and wild-type strain (WT) genome as templates, respectively. The sequence is to amplify the target gene *Piwsc1*, the resistance gene *HygB*, cross-5′-region+target gene fragment, cross-target gene+3′-region fragment, and cross-recombination fragment. (d) PCR validation of each fragment of the complementation strain (Δ*Piwsc1-co*). Lane 1: 5′-region; lane 2: *G418*; lane 3: *PtrpC*; and lane 4: *Piwsc1*+3′-region. (e) RT-PCR analysis of the transcription of *Piwsc1* in different strains with gene-specific primers wsc1–5-F/R. The *β-actin* gene in *P. italicum* is used as the reference gene. Lane marker, DL 2000 marker; Figure S2: Blue mold symptoms and disease development in Navel orange wounds inoculated with wild-type *P. italicum* at 24, 48, and 72 hpi; Figure S3: twenty-nine candidate effector genes are verified by RT-PCR. M: indicates 2000 bp DNA marker. 1–29: PITC_048860, PITC_019680, PITC_061260, PITC_045800, PITC_077450, PITC_051450, PITC_068580, PITC_014450, PITC_085860, PITC_014290, PITC_062780, PITC_013620, PITC_020870, PITC_081470, PITC_008610, PITC_047900, PITC_034160, PITC_001010, PITC_015600, PITC_005000, PITC_081180, PITC_043760, PITC_099400, PITC_097880, PITC_087410, PITC_016950, PITC_014210, PITC_079910, PITC_007270. The red asterisk “*” indicates that gene expression cannot be detected; Figure S4: Sequence alignment of three PiWSC proteins. Table S1: Gene-specific primer sequences designed for RT-PCR of 29 candidate effector proteins. * Twelve randomly selected candidate effector genes for RT-qPCR verification; Table S2: Primers for the knockout and complementation of *Piwsc1* gene. The bold letters represent the partial sequences of HygB-F primers and HygB-R primers, respectively; the underlined marks are the partial sequences of the G418-R primers; the italic letters are the partial sequences of the PtrpC-R primers; Table S3: Secreted proteins filtered sequentially from 654 to 375; Table S4: Annotation of secreted proteins in carbohydrate-active enzymes (CAZymes); Table S5: FPKM values of 375 secreted proteins in RNA-Seq; Table S6: Highly expressed small-sized and cysteine-rich secreted proteins; Table S7: Highly expressed secreted proteins belonging to known effector protein families; Table S8: Highly expressed secreted proteins with EffectorP-fungi software prediction.
