# Supplementary figures and images for: Identification of Pathogenicity-Related Effector Proteins and the Role of Piwsc1 in the Virulence of Penicillium italicum on Citrus Fruits
Source: J Fungi (Basel). 2022 Jun 20;8(6):646. doi: 10.3390/jof8060646 (PMC9224591; doi:10.3390/jof8060646)

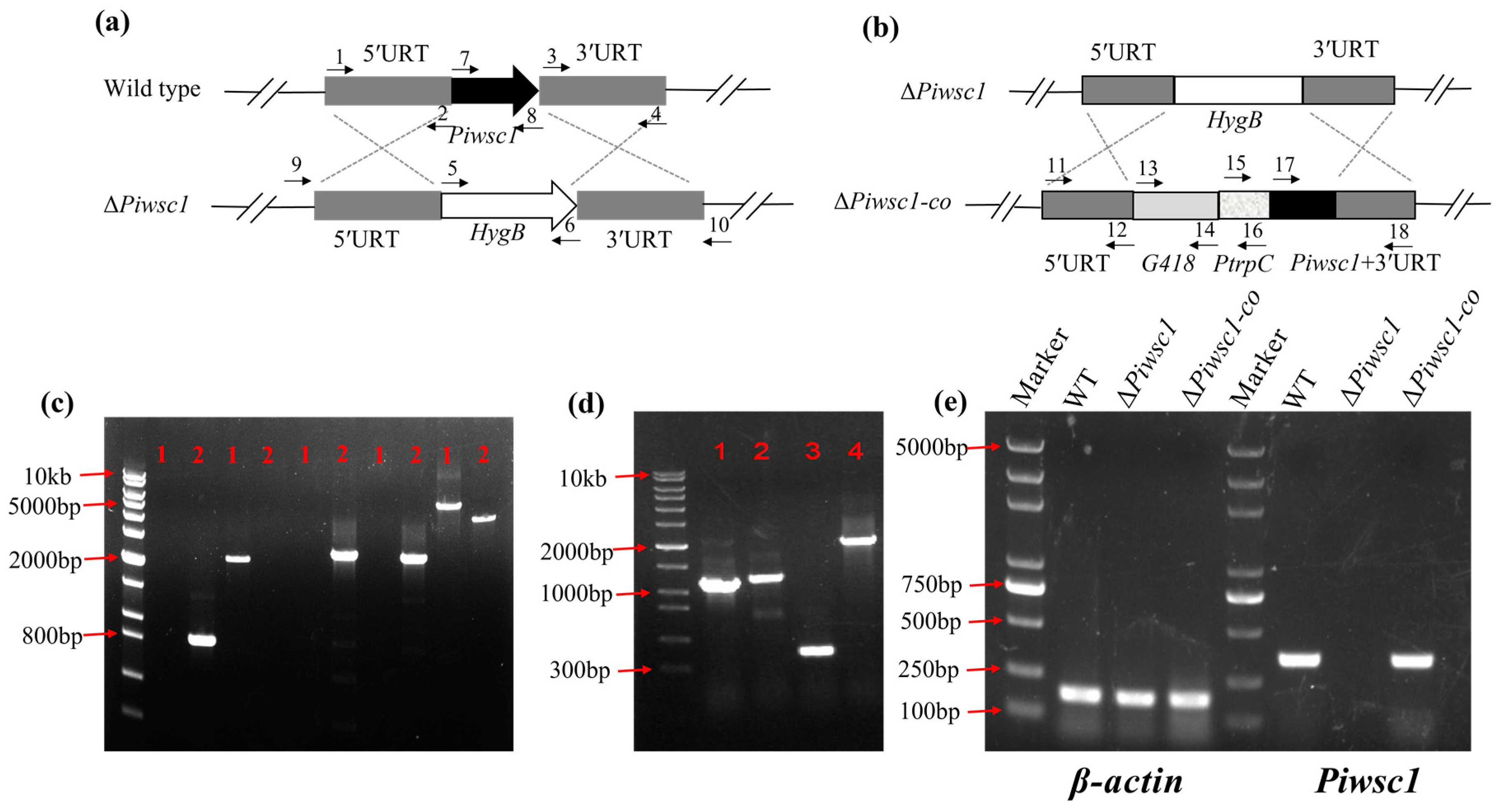

Supplement: Supplementary file 1 [file jof-08-00646-s001.zip › jof-1709786-supplementary/Supplementary data/Figure S1.tif]

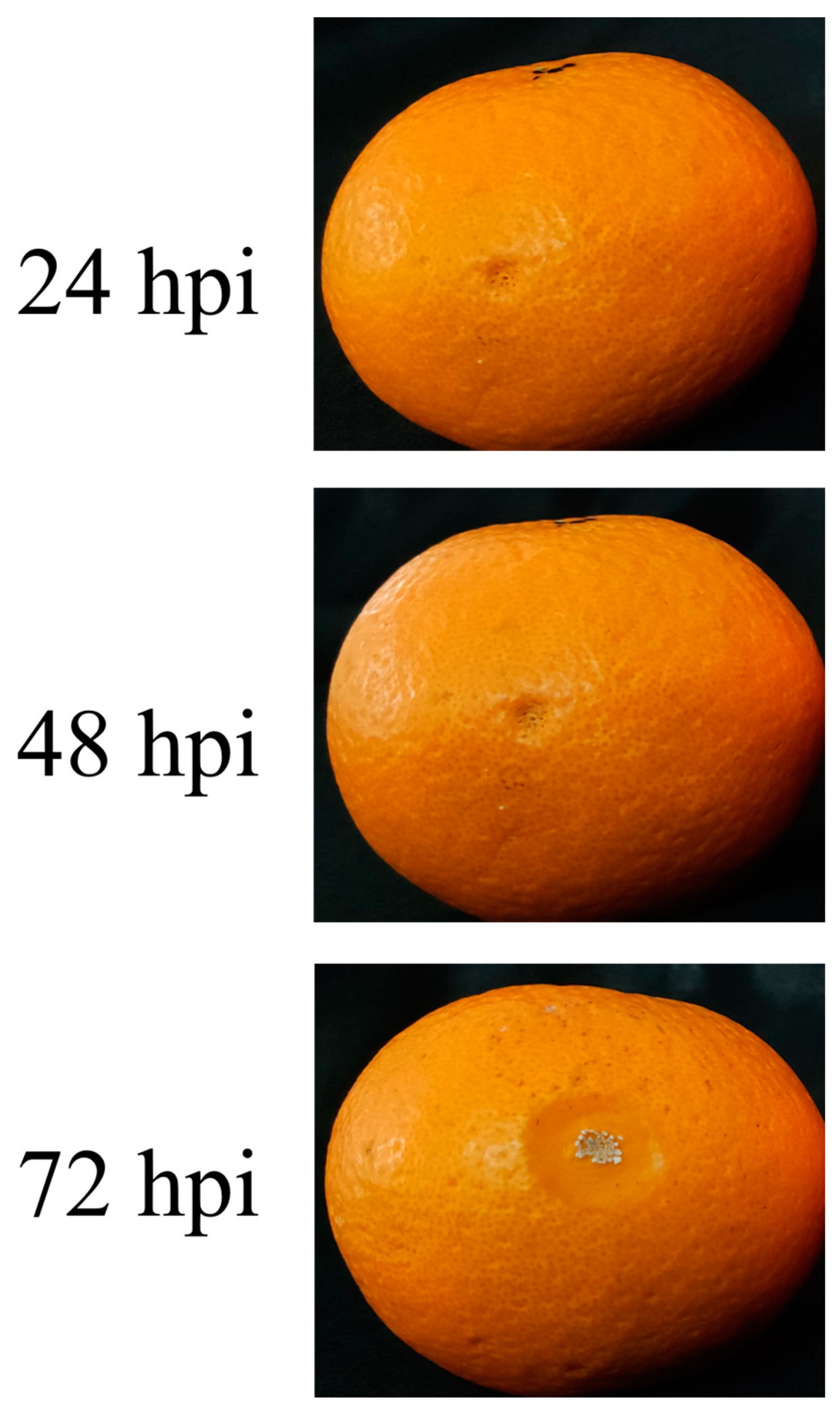

Supplement: Supplementary file 1 [file jof-08-00646-s001.zip › jof-1709786-supplementary/Supplementary data/Figure S2.tif]

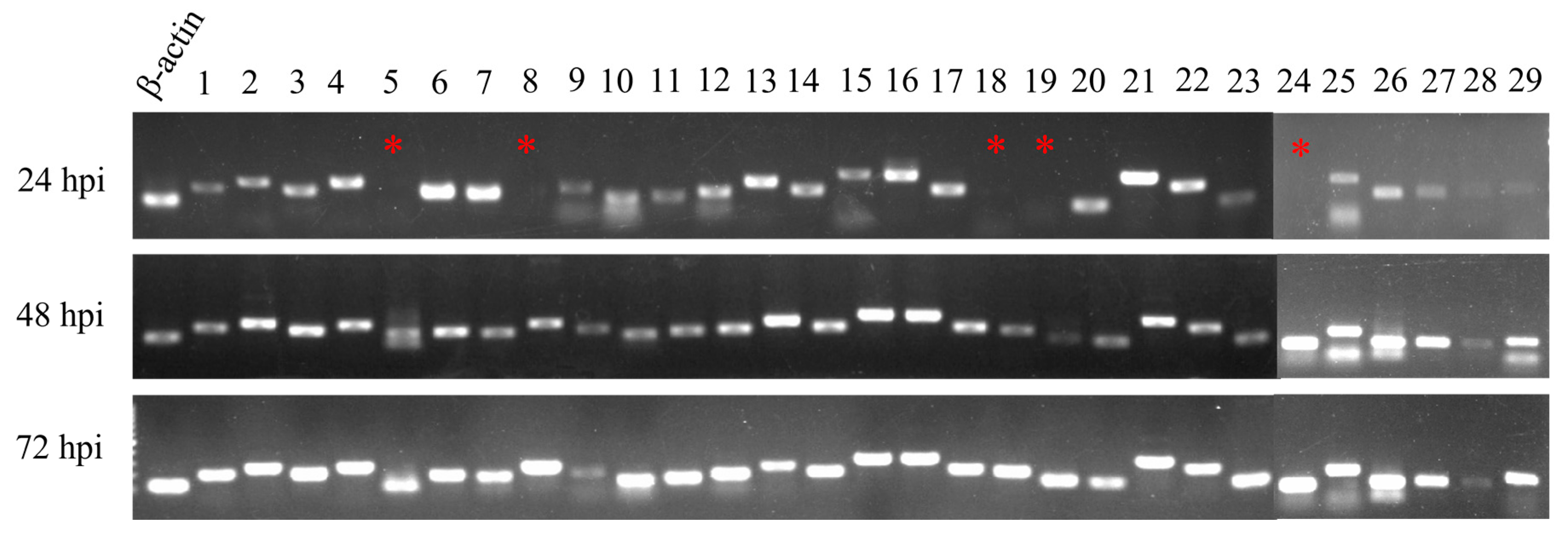

Supplement: Supplementary file 1 [file jof-08-00646-s001.zip › jof-1709786-supplementary/Supplementary data/Figure S3.tif]

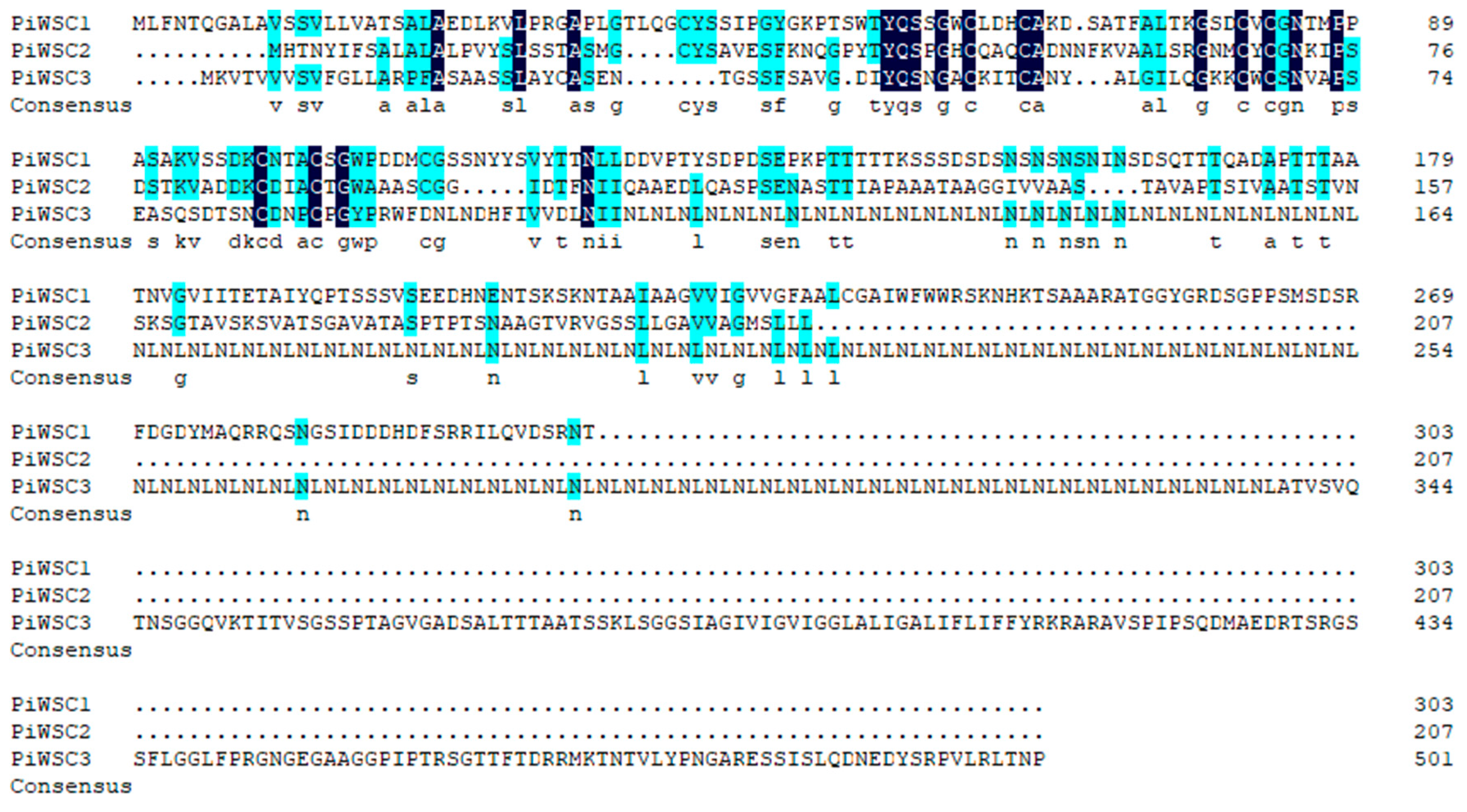

Supplement: Supplementary file 1 [file jof-08-00646-s001.zip › jof-1709786-supplementary/Supplementary data/Figure S4.tif]
